# Severity and Outcomes of Acute Malnutrition in Children Aged 0–59 Months at Guzara District Hospital, Herat: A Retrospective Study

**DOI:** 10.1002/hsr2.73342

**Published:** 2026-10-05

**Authors:** Nademah Tamkin, Uttara Partap, Matthew Leming

**Affiliations:** ^1^ Medical Department Herat University Herat Afghanistan; ^2^ University of Cambridge, Institute of Metabolic Science and MRC Metabolic Research Laboratories, Addenbrooke's Hospital Cambridge UK; ^3^ Department of Global Health and Population Boston MA USA; ^4^ Massachusetts General Hospital/Harvard Medical School Boston MA USA

**Keywords:** child health, epidemiology, inpatient department (IPD), mid‐upper arm circumference (MUAC), moderate acute malnutrition (MAM), nutrition, oedema, outpatient department (OPD), severe acute malnutrition (SAM)

## Abstract

**Background and Aims:**

Recent reports indicate a rising prevalence of acute malnutrition among children in Afghanistan. This retrospective hospital‐based study was conducted at Guzara District Hospital in Herat and aimed to investigate the severity, treatment outcomes, and monthly trends of acute malnutrition among children aged 0–59 months and potential determinants from May 2020 to March 2022.

**Methods:**

For this study, the data were collected from the hospital malnutrition treatment register, a standardized logbook in which demographic, anthropometric, treatment, and outcome data of admitted children were recorded. The study included 253 children, with malnutrition severity assessed using weight‐for‐height *Z*‐scores (WHZ), mid‐upper arm circumference (MUAC), and bilateral oedema. Information on age, sex, and location was also collected. In addition, treatment outcomes were obtained from the hospital registration book. WHZ, MUAC, age, sex, location, and oedema refer to characteristics recorded at baseline (admission). Group differences were analysed using Chi‐squared and Kruskal–Wallis tests, with Monte Carlo simulations for small counts.

**Results:**

Results showed a high burden of severe acute malnutrition (SAM). Based on WHZ, 77.9% of children were classified as SAM, and 14.68% presented with bilateral oedema. Among children older than 6 months, and among children with available MUAC data (*n* = 109), 74.3% had a MUAC < 115 mm. Moderate acute malnutrition (MAM) was identified in 11.46% using WHZ and 18.34% using MUAC. Regarding outcomes, 42.29% recovered, and 41.50% were referred to outpatient care, while 7.11% were referred to inpatient care, 3.55% defaulted, and 5.53% had missing outcome data. Treatment outcomes varied, with children aged 6–59 months and those with oedema requiring more extended care. Additionally, malnutrition cases increased in summer 2021.

**Conclusion:**

This study highlights the significant burden of SAM at Guzara Hospital, especially among children under 12 months, a seasonal rise in cases, and the association between treatment outcomes and malnutrition indicators.

## 1 Introduction

Nutrition plays a crucial role in supporting child health, survival, and development. Malnutrition is one of the most significant public health concerns the world faces today [1]. According to the WHO, malnutrition may include undernutrition (i.e., wasting, stunting, and underweight), micronutrient deficiency, obesity, and diet‐related non‐communicable chronic diseases [2].

Malnutrition is a serious global health problem, especially in low‐ and middle‐income countries among children. A global survey in 2020 showed that 149 million children under the age of five were stunted (low height for age), 45 million were wasted (low weight for height), and 38.9 million were overweight or obese [2].

Acute malnutrition continues to be a prevalent and alarming health challenge among children across Afghanistan. A 2013 Afghanistan Ministry of Public Health cross‐sectional household survey on malnutrition indicated a decrease in the prevalence of stunting and underweight, but a slow increase in wasting among children under five, compared to the 2004 malnutrition survey [3, 4, 5]. A study conducted in Faryab, Afghanistan, in 2016 among children less than 5 years old put the acute malnutrition rate at 35% [6]. To address this problem, by 2018, 3135 active health centres had been built in Afghanistan, which put about 87 percent of the population within a 2‐h distance of a health facility [7, 8, 9]. Despite these efforts, a 2018 UNICEF survey showed that 41% of children under five in Afghanistan were stunted, one of the highest rates in the world [10]. Additionally, Afghanistan is one of the most fragile countries in South Asia, globally ranking 108th among 117 countries in a 2019 Global Hunger Index and 42nd among 45 countries according to the Hunger and Nutrition Commitment Index, which is based on a government's commitment to addressing hunger and malnutrition [11, 12]. More recent developments in Afghanistan have exacerbated this problem; in an October 2021 report, the WHO claimed, “an estimated 3.2 million children under the age of 5 years are expected to suffer from acute malnutrition by the end of the year. At least 1 million of these children are at risk of dying due to SAM if they do not receive immediate treatment” [13].

The maintenance of SAM treatment services across most nutritional health facilities in Afghanistan is within SPHERE standards parameters [14]. The SPHERE standard [15] states that for the management of severe acute malnutrition, a recovery rate of > 75%, a death rate of < 10% and a defaulter rate of < 15% are indicators of good performance. Such metrics have been applied across various settings in South Asia and in Sub‐Saharan Africa. For example, in 2018, OPD‐SAM‐admitted children had average national‐level recovery rates of 83%, death rates of 0.04%, and defaulter [16] rates of 12.5%. IPD‐SAM‐admitted children had the average national level cure rates of 85%, death rates of 4%, and defaulter rates of 11% [14]. In Afghanistan specifically, a comparison of studies between 2019 and 2020 in children aged under 5 years old showed recovery rates increased from 73 percent to 86.5 percent, and the default rate declined from 26 percent to 11 percent for the Targeted Supplementary Feeding Program [17]. However, since at least 1 million children in Afghanistan are at risk of dying due to SAM, continued proper management of these children will be required [13].

Proper management of recovery facilities is critical during an active public health crisis. However, evidence from hospital‐based studies in Afghanistan remains limited, and challenges such as incomplete follow‐up and missing clinical data may affect the understanding of treatment outcomes.

In our study of children aged five and under in Herat, Afghanistan, we aimed to characterize the severity of acute malnutrition in referred patients, their outcomes, and the potentially associated factors, including sex, age, and geographical location. We conducted a retrospective analysis of treatment data from Guzara District Hospital in Herat. This work may help medical care centres in planning treatment, predicting outcomes, utilizing resources for patients, and preventing the high prevalence of malnutrition in the future.

## 2 Methods

### 2.1 Study Area and Duration

It is a retrospective hospital‐based study using de‐identified complete hospital records from May 2020 to March 2022 from the Guzara District Hospital, which provides inpatient and outpatient services for malnutrition patients in Herat, a province in western Afghanistan, including OPD‐MAM, OPD‐SAM, and IPD‐SAM. According to the nutrition department of Herat Public Health, in Herat, there are 94 outpatient and 6 inpatient malnutrition centres, including the Guzara District Hospital. In this study, districts were divided into three categories: the first category includes Guzara, the second category comprises the districts of Injil, Pashtun Zarghun, Adraskan, and Zinda Jan that border Guzara directly, and the third category encompasses all other districts that do not border Guzara directly.

### 2.2 Data Collection Procedure and Sample Size

Data were collected from the Guzara District Hospital registration book with permission from the Kabul Institutional Review Board (IRB) and local authorities. All procedures involving human participants adhered to the ethical standards set by the IRB and the Declaration of Helsinki (1964), as revised in 2013.

The dataset comprised routinely collected clinical information from all children aged 0–59 months who were admitted to Guzara District Hospital for acute malnutrition between May 2020 and March 2022. Ten duplicate records and three individuals classified as not SAM/MAM according to hospital criteria were excluded, leaving 253 children in the study. Among the variables recorded in the registration book were age, sex, district of origin, anthropometric measurements (weight, height, and mid‐upper arm circumference), oedema, and treatment results.

### 2.3 Definitions of Study

Malnutrition can be classified into three groups: acute, chronic, and mixed [18]. According to the WHO, acute malnutrition can be severe (SAM) or moderate (MAM). SAM was defined as a weight‐for‐height *Z*‐score (WHZ) < −3 or the presence of bilateral pitting oedema. In children aged 6–59 months, a mid‐upper arm circumference (MUAC) < 115 mm was also used as a criterion for SAM [19, 20]. MAM was defined as a WHZ ≥ −3 and < −2 or, in children aged 6–59 months, a MUAC ≥ 115 mm and < 125 mm [19]. In this study, the presence of any one of the relevant criteria was sufficient to classify a child as having SAM or MAM. Children without bilateral pitting oedema, with a WHZ ≥ −2 and ≤ +2 and, for those aged 6–59 months, a MUAC ≥ 125 mm, were classified as not SAM/MAM.

We also report treatment results for patients at the Guzara District Hospital. The outcomes of treatment reported in this study are: recovered (cured or fully recovered from malnutrition), defaulted (patients who refuse to continue treatment), referred to other care centres (either other inpatient centres with better facilities or outpatient centres after improvement of their condition), death, and missing data outcome. The tables present treatment outcomes according to these baseline characteristics.

### 2.4 Data Analysis

The analysis for this research project was conducted in Python. Patient demographics and malnutrition status were summarized, with particular attention to the severity of acute malnutrition in referred children and its association with sex, age, location, and treatment outcomes. Monthly totals of malnutrition cases between May 2020 and March 2022 were also presented. Missing data were reported as such; no imputation was performed. Differences between groups were assessed using Pearson's chi‐squared test for categorical variables and the Kruskal‐Wallis test for continuous variables. Analyses were univariate and bivariate, and no adjustment was conducted for other variables. For analysis with small sample sizes or low expected counts, a Monte Carlo simulation was applied. A *p*‐value < 0.05 was considered statistically significant. All analyses were performed in Jupyter Notebook (v7.2.2) within the Anaconda Navigator environment (v2.6.3).

### 2.5 Ethical Approval

Ethical approval for this study was obtained from the Kabul Institutional Review Board (IRB; approval no. B3‐0064). Additional permission was granted by the management of Herat City General Hospital and Guzara District Hospital.

As this was a retrospective study, informed consent from individual participants was not obtained. The study protocol, including the use of de‐identified data without consent, was reviewed and approved by the Institutional Review Board (IRB). Therefore, all identifiable patient information, including names, was removed prior to analysis. Only patient identification numbers and variables related to malnutrition were extracted from the Guzara Hospital registration records.

## 3 Results

### 3.1 Population Studied

Figure 1 shows the approximate geographic origins of patients referred to Guzara Hospital, where the locations have been randomly distributed within each district. The majority of patients referred to the hospital were from the district of Guzara (68%), with a higher number of patients coming from surrounding areas, although no discernible pattern was observed.

**Figure 1:**
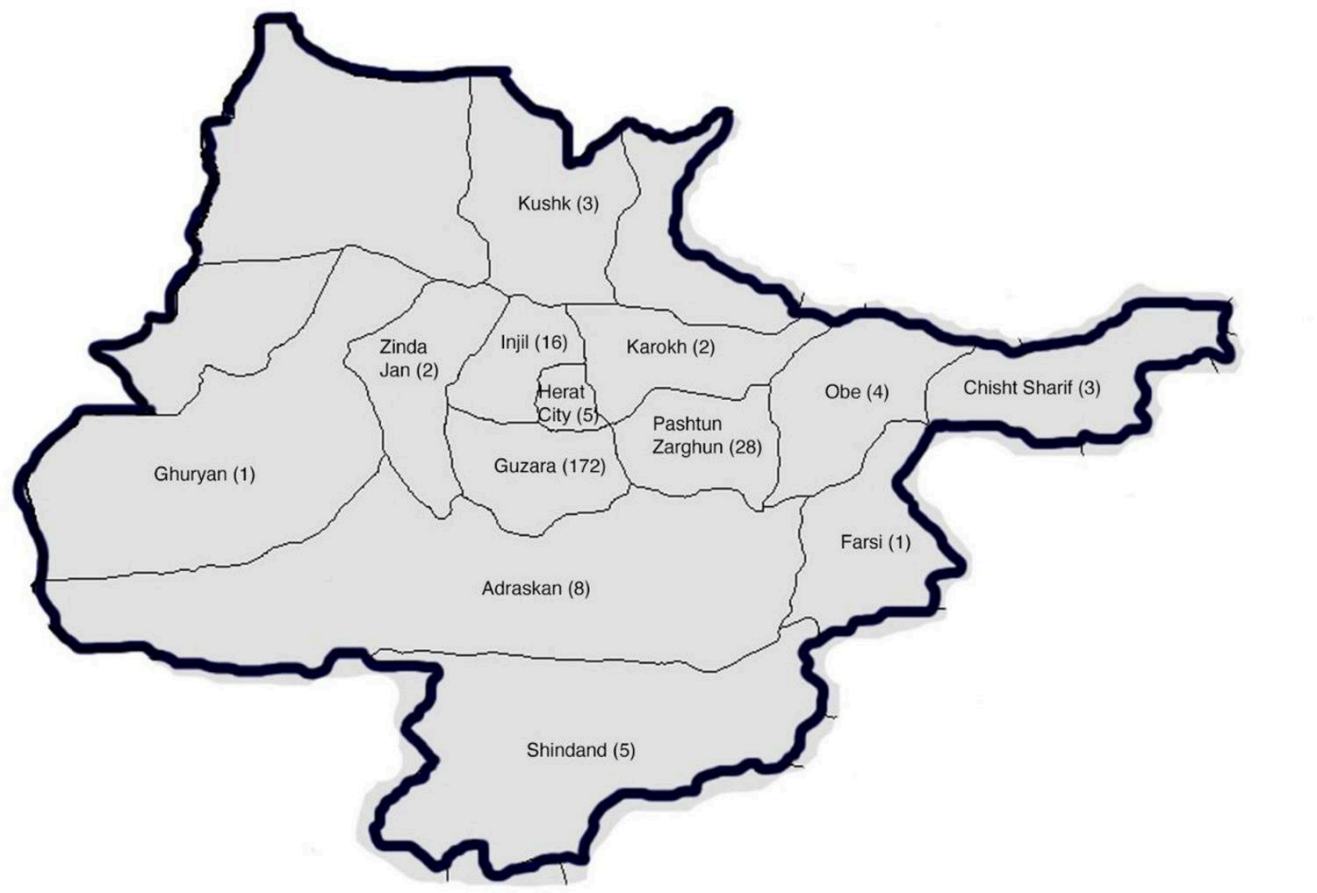
Approximate home locations of malnutrition patients of the Guzara District Hospital.

Summary statistics for 253 children are presented in Table 1. The patient population consisted of 56% females and 44% males, with ages ranging from 0 to 59 months. Acute malnutrition (moderate or severe) was prevalent in 95.2% of children under 6 months and in 85.1% of those over 6 months. Additionally, 87.3% of females and 92% of males were classified as acutely malnourished. Among children with acute malnutrition, 89.2% of children under 12 months had severe acute malnutrition.

**Table 1:** Characteristics of participants between May 2020 and March 2022 at the Guzara Hospital, Herat.

Variables	Overall (*n* = 253)	Not SAM/MAM (*n* = 27)	MAM (*n* = 29)	SAM (*n* = 197)	*p* value
Sex					**0.24**
Male	111 (43.88)	9 (8.10)	10 (9.00)	92 (82.88)	
Female	142 (56.12)	18 (12.67)	19 (13.38)	105 (73.94)	
Age					**0.007**
0– < 6 months	105 (41.50)	5 (4.0 [3.0, 4.0])	8 (3.5 [2, 4])	92 (3 [2, 4])	
6– < 12 months	98 (38.73)	12 (8.5 [7.75, 11.0])	12 (6 [6, 7])	74 (8 [7, 9.25])	
12– < 59 months	50 (19.76)	10 (12.0 [12.0, 16.5])	9 (18 [18, 18])	31 (12 [12, 18])	
WHZ median	**253 (−3.52 [−3.52, −3.05])**	**27 (−0.81 [−1.11, 0.005])**	**29 (−2.7 [−2.8, −2.39])**	**197 (−3.78 [−4.25, −3.35])**	**—**
MUAC median*	**109 (10.5 [10, 11.5]) N/A** = **39**	**8 (13 [12.875, 13.25])**	**20 (12 [11.9, 12])**	**81 (10 [10, 10.5])**	**—**
Oedema	**252 N/A** = **1**	**215 (85.31)**	**—**	**37 (14.68)**	**—**
Treatment outcome					**0.08**
Recovered	107 (42.29)	5 (4.67)	5 (4.67)	97 (90.65)	
Death	0	0	0	0	
Defaulted	9 (3.55)	3 (33.33)	2 (22.22)	4 (44.44)	
Referred to OPD‐care	105 (41.50)	15 (14.28)	16 (15.23)	74 (70.47)	
Referred to IPD‐care	18 (7.11)	0	0	18 (100)	
Missing	14 (5.53)	4 (28.57)	6 (42.85)	4 (28.57)	
Total	**253 (100)**	**27 (10.67)**	**29 (11.46)**	**197 (77.86)**	
Location					**—**
Category 1	**172 (67.98)**	**15 (8.72)**	**17 (9.88)**	**140 (81.39)**	
Guzara	172	15	17	140	
Category 2	**54 (21.34)**	**9 (16.66)**	**9 (16.66)**	**36 (66.66)**	
Pashtun Zarghun	28	4	5	19	
Zinda Jan	2	0	1	1	
Injil	16	2	2	12	
Adraskan	8	3	1	4	
Category 3	**27 (10.67)**	**3 (11.11)**	**3 (11.11)**	**21 (77.77)**	
Ghuryan	1	0	0	1	
Obe	4	0	1	3	
Chishti Sharif	3	1	0	2	
Farsi	1	1	0	0	
Herat city	5	0	0	5	
Karokh	2	0	0	2	
Kushk	3	0	1	2	
Shindand	5	0	0	5	
N/A	3	1	1	1	
Total	**253 (100)**	**27 (10.67)**	**29 (11.46)**	**197 (77.86)**	

Data are presented as *n* (%) for categorical variables and median (IQR) for non‐normally distributed continuous variables.

* Among children aged ≥ 6 months, *n* (median [IQR]).

According to WHO guidelines [19, 20], children were categorized into not SAM/MAM/MAM/SAM groups based on established criteria, including WHZ scores, mid‐upper arm circumference, and oedema. According to WHZ measurements, among patients, 10.6% were classified as not SAM/MAM (median WHZ: −0.81; IQR: −1.11 to 0.005), 11.6% as having MAM (median WHZ: −2.7; IQR: −2.8 to −2.39), and 77.8% as having SAM (median WHZ: −3.78; IQR: −4.25 to −3.35). Among the 148 children aged 6–59 months, MUAC data were missing for 39 children, leaving 109 children with available MUAC measurements. Of these 109 children, 7.3% were classified as having normal MUAC (median: 13.0 cm; IQR: 12.87–13.25), 18.3% had moderate acute malnutrition (MAM; median MUAC: 12.0 cm; IQR: 11.9–12.0), and 74.3% had severe acute malnutrition (SAM; median MUAC: 10.0 cm; IQR: 10.0–10.5). The assessment of oedema showed that 85.3% of participants had no oedema, whereas 14.7% presented with oedema and were classified as having severe acute malnutrition (SAM). All data related to the classification of the patients are characterized in Table 1.

### 3.2 Trend of Malnutrition

The results showed an increase in malnourished patients during the summer months of 2021. However, surprisingly, a decline in the number of patients at Guzara District Hospital was observed in 2020.

### 3.3 Changes in Admission Over Time

Figure 2 shows the monthly number of normal and total malnutrition cases of the Guzara hospital between May 2020 and March 2022. The SAM admission rates indicate an upward trend from January to February (10.15% of total cases) and from March through July, with a peak in June (44.67%), followed by a decline from September through October (10.15%). During this time, all Herat malnutrition centres were active. This was followed by a decline from November onwards, as winter approached.

**Figure 2:**
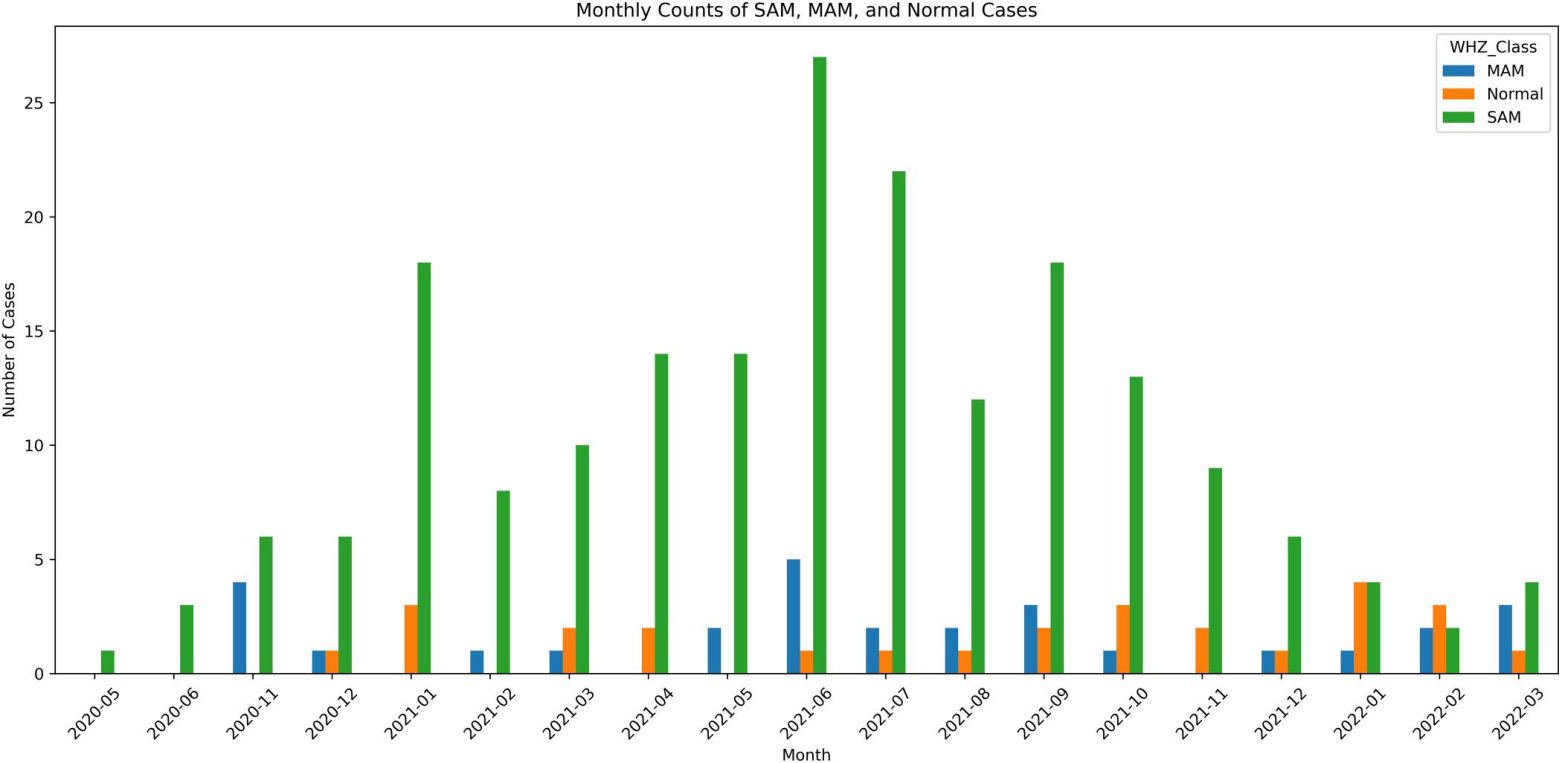
The monthly total malnutrition cases of Guzara hospital between May 2020 and March 2022, as well as the rates of moderate and severe acute malnutrition according to weight‐for‐height *Z*‐scores. Severe acute malnutrition rates increased significantly between February up to end of June in 2021.

### 3.4 End of Treatment

Of the 253 participants, 42.29% recovered, 41.50% were referred to outpatient care, 7.11% were referred to inpatient care, 3.55% defaulted, 5.53% had missing outcome data, and no deaths were recorded. Among the 197 patients with SAM, 49.24% (97/197) recovered, 37.56% (74/197) were referred to OPD care, 9.14% (18/197) were referred to IPD care, 2.03% (4/197) defaulted, and 2.03% (4/197) had missing data. Among the 29 patients with MAM, 17.24% (5/29) recovered, 55.17% (16/29) were referred to OPD care, 6.90% (2/29) defaulted, and 20.69% (6/29) had missing data. No patients with MAM were referred to IPD care.

Children aged 0– < 6 months were more likely to recover (82.86%, 87/105), whereas Children aged 6– < 59 months were more frequently referred to OPD care (70.27%, 104/148). In addition, Children with oedema were more frequently referred to OPD care (25/37, 67.57%) compared to those without oedema (80/215, 37.21%). This association was statistically significant (*p* < 0.001, Chi‐square test). The other finding was that children diagnosed with SAM based on WHZ were more frequently recovered. Although those classified as SAM based on MUAC were more often referred to outpatient care, this association was not statistically significant when the analysis was restricted to children classified as SAM or MAM based on anthropometric criteria (*N* = 226). Of the 253 participants assessed based on WHZ, MUAC, and oedema, 23 either had missing outcome data (*n* = 4) or defaulted from treatment (*n* = 9). Outcome data were available for 239 children and were missing for 14 children (Figure 3). Detailed information on treatment outcomes is presented in Tables 1 and 2.

**Figure 3:**
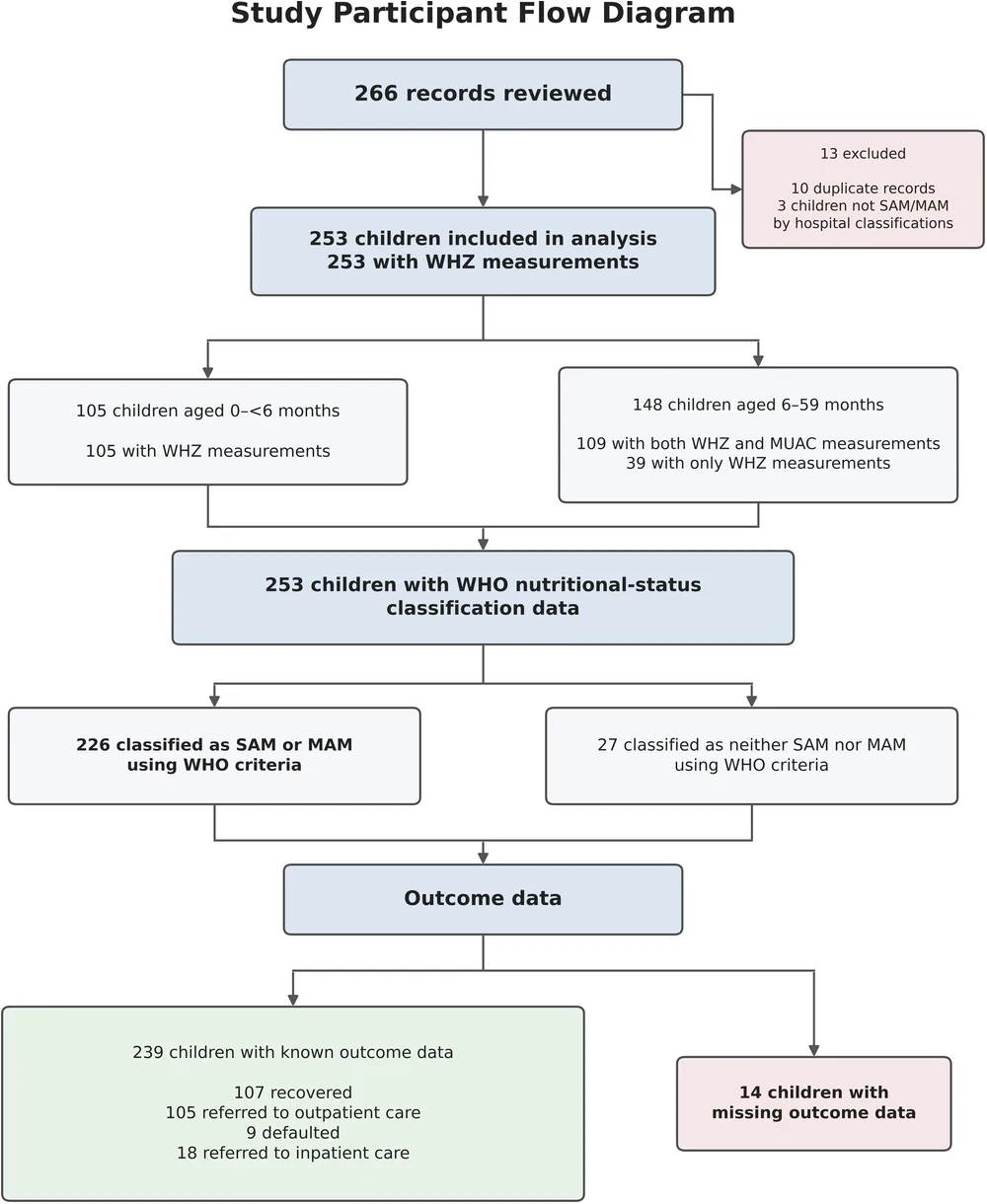
Flow diagram of study participant inclusion, nutritional‐status classification, and treatment outcomes. A total of 266 records were reviewed, of which 13 were excluded, including 10 duplicate records and 3 children who were not classified as having severe acute malnutrition (SAM) or moderate acute malnutrition (MAM) according to the hospital classification. Overall, 253 children were included in the analysis and had weight‐for‐height z‐score (WHZ) measurements. Of these, 105 were aged 0– < 6 months and 148 were aged 6–59 months; among the latter, 109 had both WHZ and mid‐upper arm circumference (MUAC) measurements and 39 had WHZ measurements only. Using WHO classification criteria, 226 children were classified as having SAM or MAM, whereas 27 were classified as neither SAM nor MAM. Outcome data were available for 239 children: 107 recovered, 105 were referred to outpatient care, 9 defaulted, and 18 were referred to inpatient care. Outcome data were missing for 14 children.

**Table 2:** Treatment outcomes of malnutrition patients and the effects of other variables on treatment outcomes.

Variables	Overall *n* (%)	Recovered *n* (%)	Referred to OPD *n* (%)	Referred to IPD *n* (%)	Missing *n* (%)	Defaulted *n* (%)	*p* value
Sex							**0.372**
Male	111 (43.87)	45 (40.54)	49 (44.14)	10 (9.01)	3 (2.70)	4 (3.60)	
Female	142 (56.13)	62 (43.66)	56 (39.44)	8 (5.63)	11 (7.75)	5 (3.52)	
Age group							**< 0.001**
0– < 6 months	105 (41.50)	87 (82.86)	1 (0.95)	5 (4.76)	8 (7.62)	4 (3.81)	
6– < 12 months	98 (38.74)	19 (19.39)	61 (62.24)	10 (10.20)	3 (3.06)	5 (5.10)	
12– < 59 months	50 (19.76)	1 (2.00)	43 (86.00)	3 (6.00)	3 (6.00)	0 (0.00)	
WHZ classification							**0.001**
SAM	197 (77.87)	97 (49.24)	74 (37.56)	18 (9.14)	4 (2.03)	4 (2.03)	
MAM	29 (11.46)	5 (17.24)	16 (55.17)	0 (0)	6 (20.69)	2 (6.90)	
Not SAM‐MAM	27 (10.67)	5 (18.52)	15 (55.56)	0 (0)	4 (14.81)	3 (11.11)	
MUAC classification							**0.554**
SAM	81 (74.3)	1 (1.23)	69 (85.18)	6 (7.40)	2 (2.47)	3 (3.70)	
MAM	20 (18.34)	0 (0.00)	18 (90.00)	1 (5.00)	1 (5.00)	0 (0.00)	
Normal	8 (7.33)	0 (0.00)	5 (62.50)	1 (12.50)	1 (12.50)	1 (12.50)	
Oedema classification							**0.001**
SAM/oedema present	37 (14.62)	2 (5.41)	25 (67.57)	4 (10.81)	4 (10.81)	2 (5.41)	
No oedema	215 (84.98)	104 (48.37)	80 (37.21)	14 (6.51)	10 (4.65)	7 (3.26)	
N/A	1 (0.40)	1 (100.00)	0 (0.00)	0 (0.00)	0 (0.00)	0 (0.00)	
Location							**0.250**
Category 1	**172 (67.98)**	**77 (44.77)**	**72 (41.86)**	**11 (6.40)**	**9 (5.23)**	**3 (1.74)**	
Guzara	172 (67.98)	77 (44.77)	72 (41.86)	11 (6.40)	9 (5.23)	3 (1.74)	
Category 2	**54 (21.34)**	**18 (33.33)**	**24 (44.44)**	**6 (11.11)**	**2 (3.70)**	**4 (7.41)**	
Pashtun Zarghun	28 (11.07)	11 (39.29)	12 (42.86)	2 (7.14)	0 (0.00)	3 (10.71)	
Zinda Jan	2 (0.79)	0 (0.00)	1 (50.00)	0 (0.00)	1 (50.00)	0 (0.00)	
Injil	16 (6.32)	6 (37.50)	8 (50.00)	2 (12.50)	0 (0.00)	0 (0.00)	
Adraskan	8 (3.16)	1 (12.50)	3 (37.50)	2 (25.00)	1 (12.50)	1 (12.50)	
Category 3	**27 (10.67)**	**12 (44.44)**	**9 (33.33)**	**1 (3.70)**	**3 (11.11)**	**2 (7.41)**	
Ghuryan	1 (0.40)	0 (0.00)	1 (100.00)	0 (0.00)	0 (0.00)	0 (0.00)	
Obe	4 (1.58)	1 (25.00)	3 (75.00)	0 (0.00)	0 (0.00)	0 (0.00)	
Chishti Sharif	3 (1.19)	2 (66.67)	1 (33.33)	0 (0.00)	0 (0.00)	0 (0.00)	
Farsi	1 (0.40)	0 (0.00)	0 (0.00)	0 (0.00)	0 (0.00)	1 (100.00)	
Herat City	5 (1.98)	2 (40.00)	1 (20.00)	0 (0.00)	2 (40.00)	0 (0.00)	
Karokh	2 (0.79)	1 (50.00)	1 (50.00)	0 (0.00)	0 (0.00)	0 (0.00)	
Kushk	3 (1.19)	2 (66.67)	0 (0.00)	0 (0.00)	1 (33.33)	0 (0.00)	
Shindand	5 (1.98)	4 (80.00)	0 (0.00)	0 (0.00)	0 (0.00)	1 (20.00)	
N/A	3 (1.19)	0 (0.00)	2 (66.67)	1 (33.33)	0 (0.00)	0 (0.00)	

## 4 Discussion

The study mainly focused on reporting the severity of acute malnutrition and associated variables among children referred to the Guzara District Hospital. More than half of the patients (54.9%) were female, and the majority (82.3%) were aged 0– < 12 months. Overall, among acute malnutrition patients, 77.87% had a −3 *Z*‐score, and among children with available MUAC, 74.31% (81/109) were classified as SAM by MUAC. Among participants, 14.68% had bilateral oedema with different degrees of oedema. Moderate malnutrition cases accounted for 11.46% (29/253) with a *Z*‐score between −2 and −3, and among the 109 children with available MUAC, 18.3% (20/109) were classified as MAM. About half were recovered, and a sizeable proportion were also referred out.

Although the high and increasing burden of acute malnutrition among children in Afghanistan has been recognized [21], to our knowledge, data examining outcomes of children with acute malnutrition and their potential predictors in the country remain scarce in the peer‐reviewed literature. Given the current burden of malnutrition in the country, the ability of facilities to accommodate patients with acute malnutrition is pivotal. Understanding recent trends in hospital admissions as well as treatment outcomes and potential predictors may help to improve planning and algorithms of care. The current study contributes data in this regard, pointing towards potentially important areas to focus on to further improve treatment outcomes.

Children under 6 months were more likely to recover from inpatient care, whereas children aged 6–59 months were more commonly referred to outpatient therapeutic programmes. After treatment, the overall rates of recovery, transfer to OPD care, death, defaulter, missing data, and transfer to other IPD‐care centres with better facilities were 42.3%, 41.5%, 0%, 3.5%, 5.5%, and 7.1%, respectively – these outcomes of treatment were significantly associated with age. Findings related to younger age and greater likelihood of discharge are somewhat in contrast to other previous literature, which generally indicates greater likelihood of recovery among older children [22], with young age being recognized as contributing to a higher risk of mortality among children with SAM [23]. Importantly, however, this literature is not fully consistent – for example, a study based in Yemen indicated no clear associations between age and time to recovery among children with SAM aged 6–59 months [24], and furthermore, data from children under 6 months remain generally more limited than for older children. Different factors may affect likelihood of recovery among infants aged 0–6 months, including prevalence of breastfeeding – which has also previously been associated with reduced time to recovery from SAM [24]. Algorithms of care for older versus younger children may also be distinct, including by setting. Together with this previous literature, our findings reinforce the potential importance of setting‐specific factors in determining outcomes, and resultant need for greater setting‐specific data when examining treatment outcomes for SAM.

The significant association between the presence of oedema and treatment outcome indicates that oedematous children were more frequently referred to outpatient therapeutic care. This may reflect clinical caution or standardized treatment protocols that prioritize follow‐up monitoring for children with oedematous forms of malnutrition. Oedema is a hallmark of SAM and may prompt extended care beyond inpatient stabilization, even when immediate complications have resolved. Prior studies based in sub‐Saharan Africa have reported better treatment outcomes among children (both in inpatient and outpatient treatment) with SAM who had oedema, hypothesized to be attributed to the perception of oedema potentially leading to improved adherence to treatment [25, 26]. These findings highlight the importance of integrated inpatient–outpatient continuity of care, especially for oedematous cases.

Children diagnosed with SAM based on WHZ were more frequently recovered. In contrast, no statistically significant difference in treatment outcomes was observed according to MUAC classification. Although MUAC and WHZ may identify partially distinct subpopulations of children with SAM, these findings do not provide evidence of a differential treatment outcome according to MUAC. The present study was not designed to evaluate treatment algorithms directly; however, the findings highlight the importance of coordinated inpatient and outpatient care and integrated management approaches for children with SAM [27].

The peak and upward trend in summer may be associated with seasonal variation in malnutrition incidence [28]. Another possible explanation may relate to broader socioeconomic conditions in Afghanistan. The increasing number of SAM admissions in recent years may also reflect improved access to or utilization of SAM treatment services in Afghanistan's poorest provinces; however, this could not be directly assessed in the present study. In contrast, a decline in the number of patients at Guzara District Hospital in 2020 was observed. A possible explanation for this reduction may be reduced health service utilization due to public fear of COVID‐19 infection [17]. Subsequently, an increase in admissions was observed in early 2021, which may reflect delayed care‐seeking following the pandemic period. Broader and interrelated determinants, including environmental determinants such as rainfall and temperature patterns, as well as community‐acquired infections, have previously been shown to be associated with time to recovery among children with acute malnutrition in settings such as Niger [29], and could likely also contribute to patterns of incidence. Although we did not have available data to assess these patterns, future analyses more robustly assessing potential environmental and food systems‐related drivers of acute malnutrition in Afghanistan may be ideal, in line with previous literature in this regard [30].

According to the SPHERE standard [19], the results of this treatment showed a lower recovery rate but acceptable levels of default and death. However, the comparison of studies between 2019 and 2020 in children under 5 years old showed that the cure rate increased from 73 percent to 86.5 percent. The defaulter rate in the Targeted Supplementary Feeding Program declined from 26 percent to 11 percent, which was brought to an acceptable level in both recovery and defaulter rates [17]. A possible reason for this is that in this study, we were unable to track participants after their referral to OPD care.

The analysis's strength lies in using multiple variables to assess the severity of malnutrition and treatment outcomes over 2 years. With most participants classified as having SAM, the findings are clinically relevant for this high‐risk group. The study identified an increase in malnourished patients during the summer of 2021. Additionally, stratifying acute malnutrition severity by age groups could help identify underlying causes in future research.

However, this study has several limitations. The major limitation was loss to follow‐up, as patients who defaulted or discontinued treatment could not be traced, which may have introduced outcome bias. These patients may have had poorer adherence, worse clinical responses, or higher complication rates compared with those who completed follow‐up, potentially leading to an overestimation of treatment success and favourable outcomes. As this was a retrospective hospital‐based study, complete tracking of patients referred to outpatient departments or other health facilities in Herat was not feasible.

Missing data were present for a small proportion of variables; however, these were handled using complete‐case analysis without imputation, which may have introduced additional bias if the missingness was not completely random. Additionally, we conducted univariate and bivariate analyses examining associations between specific factors and outcomes, but did not adjust for other potentially associated variables in these analyses. Finally, the study is subject to selection bias due to its hospital‐based design, as it only includes children admitted to Guzara District Hospital and may not be representative of all children with severe acute malnutrition in the city or broader region. Therefore, the findings may have limited generalisability beyond the study setting.

## 5 Conclusion

This study highlights a substantial burden of severe acute malnutrition among children admitted to Guzara Hospital, particularly among those under 12 months of age. A higher number of cases was observed during the summer months, which may reflect underlying environmental or socioeconomic factors. Age group and presence of oedema were significantly associated with different treatment pathways, with children aged 6–59 months and oedematous children requiring more prolonged care. Additionally, children identified with SAM by WHZ were more often recovered, reflecting differences in management patterns. These findings emphasize the potential relevance of oedema status and anthropometric criteria in treatment planning and may help inform age‐specific prevention strategies and standardized screening approaches.

## Author Contributions

**Nademah Tamkin:** conceptualization, investigation, writing – original draft, methodology, validation, visualization, formal analysis, data curation, resources. **Uttara Partap:** writing – review and editing. **Matthew Leming:** conceptualization, methodology, validation, visualization, project administration, supervision.

## Funding

The authors have nothing to report.

## Conflicts of Interest

The authors declare no conflicts of interest.

## Author Declaration

All authors have read and approved the final version of the manuscript. Nademah Tamkin, as corresponding author, had full access to all of the data in this study and takes complete responsibility for the integrity of the data and the accuracy of the data analysis.

## Transparency Statement

The Corresponding author (Nademah Tamkin) affirms that this manuscript is an honest, accurate, and transparent account of the study being reported; that no important aspects of the study have been omitted; and that any discrepancies from the study as planned (and, if relevant, registered) have been explained.

## Data Availability

The data that support the findings of this study may be available on request from the corresponding author, pending applicable ethical and other regulatory approvals. The data are not publicly available due to privacy and ethical restrictions.
